# Reviewed article: Lopes MS, Silva VMSM, Ferreira NL, Carvalho MCRC, Freitas PP, Lopes ACS. Adherence to dietary practices recommended by the Dietary Guidelines for the Brazilian Population among people with obesity: baseline of a community trial carried out at the Health Fitness Program in Belo Horizonte, state of Minas Gerais, Brazil, 2022-2023. Epidemiol Serv Saude. 2025:34;e20240287.

**DOI:** 10.1590/S2237-96222025v34e20240287.b

**Published:** 2025-04-28

**Authors:** Raysa Alvarez

**Affiliations:** FOUSP

**Table:** 

Rating	Excellent	Good	Average	Below average	Poor
Interest		✓			
Quality			✓		
Originality			✓		
Overall			✓		

**Table:** 

Questionnaire	Yes	No	Not applicable
Does the manuscript contain new and significant information to justify publication?	✓		
Does the Abstract (Summary) clearly and accurately describe the content of the article?	✓		
Is the problem significant and concisely stated?	✓		
Are the methods described comprehensively?	✓		
Are the interpretations and conclusions justified by the results?	✓		
Is adequate reference made to other work in the field?	✓		

## Manuscript Structure:

Length of article is: Adequate

Number of tables is: Adequate

Number of figures is: Adequate

Please state any conflict(s) of interest that you have in relation to the review of this paper (state “none” if this is not applicable): None

## Comments

Prezado(a) autor(a),

Agradeço pela oportunidade de revisar seu artigo, que aborda um tema relevante e atual sobre a adesão às práticas alimentares recomendadas pelo Guia Alimentar entre usuários com obesidade no Programa Academia da Saúde em Belo Horizonte.

Após uma análise cuidadosa, gostaria de compartilhar algumas considerações e sugestões que podem aprimorar a clareza e a robustez do seu trabalho:

1. Processo de Seleção da Amostra: Recomendo incluir um diagrama de fluxo para esclarecer o processo de seleção da amostra. Isso ajudaria os leitores a entender melhor como os participantes foram escolhidos.

2. Coleta de Dados: Embora mencione o teste “Como está a sua alimentação?”, seria útil descrever o instrumento de coleta de dados, incluindo informações sobre sua validação e confiabilidade. Essa informação é crucial para fortalecer a credibilidade dos resultados.

2.1) Adicionalmente, quando mencionar a escala utilizada, é importante incluir detalhes sobre sua validação e confiabilidade para dar mais suporte às suas conclusões.

3. Treinamento da Equipe: Embora você tenha destacado o treinamento da equipe, seria interessante incluir uma seção sobre como os vieses foram minimizados e quais medidas foram adotadas para garantir a validade interna do estudo.

4. Apresentação de Tabelas e Gráficos: Notei que as tabelas e gráficos apresentam algumas inconsistências, como problemas com cabeçalhos, unidades de medida, qualidade das imagens e textos cortados. Uma revisão nesse sentido pode melhorar a compreensão e a apresentação dos dados.

Fico à disposição.

